# Supplementary material for: Consumer Perspectives on Anxiety Management in Australian General Practice
Source: Int J Environ Res Public Health. 2022 May 7;19(9):5706. doi: 10.3390/ijerph19095706 (PMC9103805; doi:10.3390/ijerph19095706)
Supplement: Supplementary file 1 [file ijerph-19-05706-s001.zip › ijerph-1654519-supplementary.pdf]

Introduction

Thank you for your interest in our survey.

The following page contains details about the project, and the information you need to know as a participant.

Australian National University

Participant Information Sheet

Researcher

The primary investigator for this research is Ms Erin Parker. Ms Parker is a Doctor of Philosophy (PhD) candidate in Clinical Psychology, in the Research School of Psychology, College of Health and Medicine at the Australian National University (ANU). Ms. Parker is supervised by Dr Michelle Banfield, a Senior Research Fellow at the Centre for Mental Health Research, in the Research School of Population Health, College of Health and Medicine at the ANU.

Project Title: Consumer perspectives on help-seeking and treatment of anxiety in primary care

General Outline of the Project

- **Description and Methodology**: The objective of this project is to investigate the treatment of anxiety in Australian primary care, from the perspective of people who receive that treatment. This aim will be achieved by conducting anonymous online surveys of people who have sought help for anxiety from their general practitioner (GP). This study is part of a larger project that aims to investigate the management of anxiety and make recommendations for improving care.

- **Participants:** This study aims to survey adult Australians (aged 18 years and above) who have seen their GP for anxiety in the past five years. Approximately 200 people will be recruited.

- **Use of data and feedback:** Results from the survey will be prepared for publication and presentation at mental health conferences, and will also be included as part of the primary investigator's PhD thesis. A plain language summary of results will be made available online here and through a shared dropbox folder that does not require login information to be viewed. The website address and link to the folder will be provided again at the conclusion of the survey.

Participant Involvement

- **Voluntary participation & withdrawal:** Participation in this research is voluntary, and you may withdraw at any point before you submit your responses to the researcher at the end of the survey. There are no consequences for withdrawing from the study, and you do not need to provide a reason for doing so. If you decide to withdraw from the survey, any information you have already provided will be destroyed and not used in the research. You may also choose not to answer

certain questions as you complete the survey.

- **What does participation in the research entail?** You will be asked to complete an anonymous online survey about your experiences of seeking help for anxiety from your GP. The survey will ask questions about your general experience of seeking help (e.g., what you wanted, whether your needs were met), the type of treatment or support you were offered, and any suggestions you have for improvements. You will also be asked questions about your anxiety, such as any symptoms you are currently experiencing, whether you have ever received a diagnosis. You will also be briefly asked about any current symptoms of depression and stress, and any impacts of COVID-19 on your experience in primary care.

- **Location and duration:** You will be asked to complete a single survey, which can be done online from a location of your choosing. The survey will take approximately 15-30 minutes to complete.

- **Risks:** We anticipate that answering questions about your experience of seeking help may cause discomfort or distress, particularly if you have had a negative experience. This may also be true for answering questions about the impact of COVID-19. If you feel distressed at any point, you may contact the services below for further support. Both services provide telephone counselling as well as online chat support.

  Lifeline
  Phone counselling (24/7): 13 11 14
  Web chat (7pm – 12am, 7 days): Lifeline Online Chat
  Website: www.lifeline.org.au

  Beyond Blue
  Phone counselling (24/7): 1300 224 636
  Web chat (3pm – 12am, 7 days): Beyond Blue Online Chat
  Website: www.beyondblue.org.au

  Additional resources are available at headtohealth.gov.au/covid-19-support/covid-19. You may also contact the primary researcher via email (erin.parker@anu.edu.au) to discuss any concerns you have about the survey.

- **Benefits:** We expect that this research will increase understanding of consumer experiences and priorities for anxiety care. This information will be combined with information gathered from the research literature, and a future study to be conducted with GPs, to hopefully improve the care of anxiety in Australia.

Exclusion Criteria
This study will not include people who saw their GP primarily for post-traumatic stress disorder or obsessive compulsive disorder. The reason for this is that both of these conditions are no longer classified as anxiety disorders, and instead fall into their own, distinct categories. People who saw their GP for anxiety when they were under 18 years will also be excluded.

Confidentiality
Information will be kept confidential as far as the law allows. Surveys are anonymous to protect confidentiality. You will be asked to provide demographic information such as your age bracket, gender, ethnicity, and the State or Territory in which you sought treatment. The survey will not ask for any other information that could potentially be used to identify you. To protect your confidentiality, please do not include any identifying information (e.g.. your name) in response to open-ended questions. If you accidentally identify yourself, this information will be deleted once the data is downloaded. Research data will be presented in aggregate form and individual responses will not be reported in full. Information provided in response to open-ended questions may be used to support numerical data (i.e., as a quote) but will not be linked with your responses to other questions.

Privacy Notice

In collecting your personal information within this research, the ANU must comply with the Privacy Act 1988. The ANU Privacy Policy is available at https://policies.anu.edu.au/ppl/document/ANUP_010007. The information you provide about your healthcare experience will not be associated with any information that would allow someone to identify you.

Data Storage

- **Where:** Data will be stored on secure, password protected computers at the ANU, in folders that are only accessible to the research team.
- **How long:** Data will be stored for a period of at least five years from the date of any publication arising from the research. Following completion of Ms Parker's PhD, the data will continue to be stored at the ANU by Dr Banfield or the Head of the Research School of Psychology.
- **Handling of data following the required storage period:** Following the required storage period, data will be archived at the Research School of Psychology, at the ANU.

Queries and Concerns

- **Contact details for more information:** If you have any questions about this research, please direct them to the primary investigator.

**Primary Investigator**	**Supervisor / Co-Investigator**
Ms Erin Parker	Dr Michelle Banfield
erin.parker@anu.edu.au	michelle.banfield@anu.edu.au

- **Contact details if in distress:**

Lifeline

Phone counselling (24/7):	13 11 14
Web chat (7pm – 12am, 7 days):	Lifeline Online Chat
Website:	www.lifeline.org.au

Beyond Blue

Phone counselling (24/7):	1300 224 636
Web chat (3pm – 12am, 7 days):	Beyond Blue Online Chat
Website:	www.beyondblue.org.au

Ethics Committee Clearance:

The ethical aspects of this research have been approved by the ANU Human Research Ethics Committee (Protocol 2019/910). If you have any concerns or complaints about how this research has been conducted, please contact:

Ethics Manager
The ANU Human Research Ethics Committee
The Australian National University
Telephone: +61 2 6125 3427
Email: Human.Ethics.Officer@anu.edu.au

Please click here to open this sheet in a new window if you would like to refer back to it while you complete your survey.

If you would like to refer back to the Information Sheet while you complete your survey, please click here to open it in a new window.

If you would like to withdraw from the survey, you can do so by closing your browser at any stage prior to submitting your responses.

Please click "next" to begin.

Inclusion Criteria

Do you live in Australia?

- ○ Yes
- ○ No

Since becoming an adult (18 years old), have you ever been to see a general practitioner (GP) in Australia for help with anxiety?

- ○ Yes
- ○ No

Thank you for your interest in participating.

Our study focuses specifically on the experience of adult Australians who have sought help for anxiety from a general practitioner (GP). You have told us that you do not live in Australia, or have not seen a GP about your anxiety since being 18 years old.

However, if you have seen any Australian health professional for help with anxiety and have suggestions about improving the standard of care, please leave a response in the box below.

[ ]

Part 1: Decision to Seek Help

The first part of the survey will ask about your experience with anxiety and deciding to seek help, including any barriers you faced.

If you do not feel comfortable answering any of the questions, you may leave them blank.

To begin, please tell us a bit about your experience with anxiety. You can write about any aspect of this experience you like (e.g., your symptoms, the impact anxiety has had on you).

[ ]

What made you decide to seek help? Select all that apply.

- ☐ My symptoms got too severe for me to handle
- ☐ Others encouraged me to seek help
- ☐ I found out where to go to get help
- ☐ ________ Other reason not listed above (please specify)

Were there any barriers that made help-seeking difficult? Select all that apply.

- ☐ No barriers
- ☐ I could not afford to get help
- ☐ I was afraid to ask for help
- ☐ I could not easily access services (e.g., due to location)
- ☐ I did not know where or how to get help
- ☐ ________ Other (please specify)

Was your general practitioner (GP) the first health professional you saw about anxiety?

- ○ Yes

- ○ No
- ○ Unsure

Who did you see, or where did you seek support, before going to see your GP? Select all that apply.

- ☐ Psychologist
- ☐ Psychiatrist
- ☐ Hospital
- ☐ Counsellor
- ☐ ________ Other (please specify)

What made you decide to seek help from your GP specifically? Select all that apply.

- ☐ Recommended by other health professional
- ☐ Recommended by family member or friend
- ☐ Information found online
- ☐ Easy to access
- ☐ Unsure
- ☐ ________ Other (please specify)

Please tell us a bit about what you were expecting when you first saw your GP.

Were you looking for any particular treatment? Select all that apply.

- ☐ No, I was not looking for anything specific
- ☐ No, just general advice or information
- ☐ Yes, medication
- ☐ Yes, referral for psychological therapy
- ☐ ________ Yes, other (please specify)

Please tell us anything else you would like to say about the decision to seek help, or your expectations of your GP.

Part 2a: First Appointment

Thank you for telling us about your experience with anxiety and the decision to seek help.

The rest of the survey will ask specifically about your experience with your GP.

The next set of questions are about when you **first** saw your GP. Please answer them as best you can.

When did you **first** seek help from a GP for anxiety?

- ○ Before 2015
- ○ 2015
- ○ 2016
- ○ 2017
- ○ 2018
- ○ 2019
- ○ 2020
- ○ Unsure

Was this GP in an urban or rural/remote area?

- ○ Urban area (major cities of Australia)
- ○ Rural and remote area (all areas outside major cities)
- ○ Unsure

Please select the State or Territory where the GP was located.

- ○ ACT
- ○ NSW
- ○ NT

- QLD
- SA
- TAS
- VIC
- WA

Please indicate how much you agree with the following statements based on your **first** experience with a GP.

	Strongly disagree	Somewhat disagree	Neutral	Somewhat agree	Strongly agree
My doctor seemed to have good knowledge about anxiety	○	○	○	○	○
My doctor seemed to have good knowledge about treatment options for anxiety	○	○	○	○	○
My doctor explained things in a way I could understand	○	○	○	○	○
My doctor listened carefully to me	○	○	○	○	○
My doctor showed respect for what I had to say	○	○	○	○	○
My doctor spent enough time with me	○	○	○	○	○
I felt safe with my doctor when I went to them about anxiety	○	○	○	○	○

Please indicate how much you agree with the following statements based on your **first** experience.

	Strongly disagree	Somewhat disagree	Neutral	Somewhat agree	Strongly agree
I was given information about anxiety (e.g., verbally, information sheets)	○	○	○	○	○
I was given information about the different treatments available for anxiety	○	○	○	○	○
I was given as much information as I wanted about how I could manage my anxiety	○	○	○	○	○
My doctor asked about my preferences for treatment	○	○	○	○	○
I felt I could refuse a specific type of treatment	○	○	○	○	○

At this **first** appointment, what treatments did your GP offer? Select all that apply.

- [ ] None
- [ ] Lifestyle advice (e.g., advice to change eating, sleeping, or exercise patterns)
- [ ] Referral to a psychologist
- [ ] Referral to a psychiatrist
- [ ] Referral to self-help therapy program (e.g., online, workbook)
- [ ] Counselling or therapy (provided by the GP themselves)

- [ ] Medication - long term (taken daily and takes a few weeks to work; please specify) ________
- [ ] Medication - short term (taken when you feel particularly anxious and works immediately; please specify) ________
- [ ] ________ Other (please specify)

Did your GP complete a Mental Health Care Plan (also called a Mental Health Treatment Plan) with you?

- ○ Yes
- ○ No
- ○ Unsure

After you **first** sought help, did you receive any of the treatments your GP recommended?

- [ ] Yes
- [ ] ________ No - I chose not to access treatment (please specify)
- [ ] No - There were barriers that prevented me from accessing treatment (please specify) ________

Please tell us about the treatment you received and who it was provided by. Select all that apply.

	Treatment Received (select)	Treatment Provider				
		GP	Psychologist	Psychiatrist	Nurse	Other
Self-help therapy program	☐	☐	☐	☐	☐	☐
Counselling or psychological therapy	☐	☐	☐	☐	☐	☐
Medication (please specify) ________	☐	☐	☐	☐	☐	☐
Other (please specify) ________	☐	☐	☐	☐	☐	☐

Please indicate how much you agree with the statements below.

	Strongly disagree	Somewhat disagree	Neutral	Somewhat agree	Strongly agree
The treatment I received improved my anxiety symptoms	○	○	○	○	○
The treatment I received improved my quality of life	○	○	○	○	○

Were there any problems with the treatment you received? (E.g., side effects, financial cost, treatment was ineffective)

[ ]

Did you seek support elsewhere for your anxiety? (E.g., online, health professional other than who my GP recommended, friends and family)

- ○ No
- ○ [ ] Yes (please specify)

Please indicate how much you agree with each of the statements below based on your **first** experience of seeking help from a GP.

	Strongly disagree	Somewhat disagree	Neutral	Somewhat agree	Strongly agree
Overall, I was satisfied with the experience of seeking help from my GP	○	○	○	○	○
Overall, I felt my needs were met by my GP	○	○	○	○	○

Is there anything else you would like to say about your **first** experience with your GP?

[ ]

Part 2b: Last 12 Months

Thank you for your responses.

The next block will ask you similar questions about your recent experiences in the **last 12 months**. Please answer as best you can.

Thank you for your responses.

The next set of questions will ask about treatment preferences, and whether you have seen multiple GPs for anxiety.

In the **last 12 months**, did you go to your GP about anxiety?

- Yes
- No
- Unsure

In the **last 12 months**, was your GP in an urban or rural/remote area?

- Urban area (major cities of Australia)
- Rural and remote area (all areas outside major cities)
- Unsure

Please select the State or Territory where the GP was located.

- ACT
- NSW
- NT
- QLD
- SA
- TAS
- VIC
- WA

Please indicate how much you agree with the following statements based on the **past 12 months**. If you have seen multiple GPs in this period, please answer as best you can based on your overall experience.

	Strongly disagree	Somewhat disagree	Neutral	Somewhat agree	Strongly agree
My doctor seemed to have good knowledge about anxiety	○	○	○	○	○
My doctor seemed to have good knowledge about treatment options for anxiety	○	○	○	○	○
My doctor explained things in a way I could understand	○	○	○	○	○
My doctor listened carefully to me	○	○	○	○	○
My doctor showed respect for what I had to say	○	○	○	○	○

	Strongly disagree	Somewhat disagree	Neutral	Somewhat agree	Strongly agree
My doctor showed respect for what I had to say	○	○	○	○	○
My doctor spent enough time with me	○	○	○	○	○
I felt safe with my doctor when I went to them about anxiety	○	○	○	○	○

Please indicate how much you agree with the following statements based on the **past 12 months**. If you have seen multiple GPs in this period, please answer as best you can based on your overall experience.

	Strongly disagree	Somewhat disagree	Neutral	Somewhat agree	Strongly agree
I was given information about anxiety (e.g., verbally, information sheets)	○	○	○	○	○
I was given information about the different treatments available for anxiety	○	○	○	○	○
I was given as much information as I wanted about how I could manage my anxiety	○	○	○	○	○
My doctor asked about my preferences for treatment	○	○	○	○	○
I felt I could refuse a specific type of treatment	○	○	○	○	○

In the **last 12 months**, what treatments did your GP **offer**? Select all that apply.

- ☐ None
- ☐ Lifestyle advice (e.g., advice to change eating, sleeping, or exercise patterns)
- ☐ Referral to a psychologist
- ☐ Referral to a psychiatrist
- ☐ Referral to self-help therapy program (e.g., online, workbook)
- ☐ Counselling or therapy (provided by the GP themselves)
- ☐ Medication - long term (taken daily and takes a few weeks to work; please specify) ________
- ☐ Medication - short term (taken when you feel particularly anxious and works immediately; please specify) ________
- ☐ ________ Other (please specify)

Please indicate which treatments (if any) you received for anxiety in the **last 12 months.** Select all that apply.

	Treatment Received	Treatment Provider				
	(select)	GP	Psychologist	Psychiatrist	Nurse	Other
I did not receive treatment in the last 12 months	☐	☐	☐	☐	☐	☐
Self-help therapy program	☐	☐	☐	☐	☐	☐
Counselling or psychological therapy	☐	☐	☐	☐	☐	☐

	Treatment Received (select)	Treatment Provider				
		GP	Psychologist	Psychiatrist	Nurse	Other
Medication (please specify) ☐	☐	☐	☐	☐	☐	☐
Other (please specify) ☐	☐	☐	☐	☐	☐	☐

Please indicate how much you agree with the statements below about your treatment in the **last 12 months**.

	Strongly disagree	Somewhat disagree	Neutral	Somewhat agree	Strongly agree
The treatment I received improved my anxiety symptoms	○	○	○	○	○
The treatment I received improved my quality of life	○	○	○	○	○

Were there any problems with the treatment you received in the **last 12 months**? (E.g., side effects, financial cost, treatment was ineffective)

Please indicate how much you agree with each of the statements below based on your experience in the **past 12 months**.

	Strongly disagree	Somewhat disagree	Neutral	Somewhat agree	Strongly agree
Overall, I was satisfied with the experience of seeking help from my GP	○	○	○	○	○
Overall, I felt my needs were met by my GP	○	○	○	○	○

Is there anything else you would like to say about your experience in the **past 12 months**?

Thank you for telling us about your recent experience.

The next set of questions will ask about treatment preferences, and whether you have seen multiple GPs for anxiety.

Part 2c: Treatment Preferences

Do you **currently** have a Mental Health Care Plan (also called a Mental Health Treatment Plan)?

- ○ Yes
- ○ No
- ○ Unsure

Do you have a regular GP?

- ○ Yes
- ○ No

Since becoming an adult, have you seen more than one GP for anxiety?

- ○ Yes
- ○ No

What were your reasons for changing GPs? Select all that apply.

- ☐ Dissatisfied with GP
- ☐ I moved to another location
- ☐ GP moved/retired/went on leave
- ☐ I do not have a regular GP
- ☐ ________ Other (please specify)

Please tell us anything else you would like to say about your reasons for changing GPs.

[ ]

When considering treatment for your anxiety, which of the following things are important to you? Select all that apply.

- ☐ How much it costs
- ☐ Potential side effects
- ☐ How quickly it works
- ☐ How well it works
- ☐ How easy it is to access
- ☐ ________________ Other (please specify)

Please drag to rank your answers from most to least important

How much it costs

Potential side effects

How quickly it works

How well it works

How easy it is to access

${q://QID51/ChoiceTextEntryValue/9}

Is there anything else you would like to say about your preferences for treatment?

Part 3: Symptoms and Diagnosis

Thank you for giving us some information about your experiences with help-seeking and treatment.

The next set of questions are about your current anxiety symptoms and any diagnoses you have been given.

We are collecting this information because it helps us understand the experiences of people with different types of anxiety. It also helps us know how well our results apply to

the wider population. It does not matter whether you have been given a diagnosis or not, we are still interested in your experience.

If you do not feel comfortable answering certain questions, you can select "prefer not to say" or leave the answer blank.

Please read each statement and indicate how much the statement applied to you **over the past week**. There are no right or wrong answers. Do not spend too much time on any statement.

	Never	Sometimes	Often	Almost Always
I found it hard to wind down	○	○	○	○
I was aware of dryness of my mouth	○	○	○	○
I couldn't seem to experience any positive feeling at all	○	○	○	○
I experienced breathing difficulty (e.g., excessively rapid breathing, breathlessness in the absence of physical exertion)	○	○	○	○
I found it difficult to work up the initiative to do things	○	○	○	○
I tended to over-react to situations	○	○	○	○
I experienced trembling (e.g., in the hands)	○	○	○	○
I felt that I was using a lot of nervous energy	○	○	○	○
I was worried about situations in which I might panic and make a fool of myself	○	○	○	○
I felt that I had nothing to look forward to	○	○	○	○
I found myself getting agitated	○	○	○	○
I found it difficult to relax	○	○	○	○
I felt down-hearted and blue	○	○	○	○
I was intolerant of anything that kept me from getting on with what I was doing	○	○	○	○
I felt I was close to panic	○	○	○	○
I was unable to become enthusiastic about anything	○	○	○	○
I felt I wasn't worth much as a person	○	○	○	○
I felt that I was rather touchy	○	○	○	○
I was aware of the action of my heart in the absence of physical exertion (e.g., sense of heart rate increase, heart missing a beat)	○	○	○	○
I felt scared without any good reason	○	○	○	○
I felt that life was meaningless	○	○	○	○

We are also interested in the impact of COVID-19 on your experience in the past few months.

As with other questions in the survey, you may choose not to answer if you prefer.

Please tell us how COVID-19 has affected any **treatment** you are receiving

Compared to previously, how likely are you to seek help for your anxiety?

- More likely
- Less likely
- Unchanged

Please tell us anything else you would like to say about the impact of COVID-19.

Thank you for answering the previous questions. If you feel distressed or are concerned, please contact your healthcare provider or the support services below.

Lifeline

Phone counselling (24/7):	13 11 14
Web chat (7pm – 12am, 7 days):	www.lifeline.org.au/get-help/online-services/crisis-chat
Website:	www.lifeline.org.au

Beyond Blue

Phone counselling (24/7):	1300 224 636
Web chat (3pm – 12am, 7 days):	www.online.beyondblue.org.au/OutOfHours#/chat/questions1
Website:	www.beyondblue.org.au

Please click "next" to continue with the survey. The next two questions are about diagnosis.

Have you ever been given a mental health diagnosis? Please select all that apply.

- ☐ I have never been given a diagnosis
- ☐ Generalised Anxiety Disorder
- ☐ Social Anxiety Disorder (Social Phobia)
- ☐ Agoraphobia
- ☐ Panic Disorder
- ☐ Specific Phobia (e.g., animals, flying)
- ☐ Obsessive Compulsive Disorder
- ☐ Posttraumatic Stress Disorder
- ☐ Adjustment Disorder
- ☐ Major Depressive Disorder
- ☐ Substance Use Disorder
- ☐ Bipolar Disorder
- ☐ Psychotic Disorder (e.g., Schizophrenia)
- ☐ Eating Disorder
- ☐ Personality Disorder
- ☐ Autism Spectrum Disorder (incl. Asperger's Syndrome)
- ☐ Unsure or prefer not to say
- ☐ Other (please specify) ________

Please tell us who gave you the diagnosis/es and in what year.

	Diagnosed by					Year
	GP	Psychologist	Psychiatrist	Other	Unsure	(enter)
Generalised Anxiety Disorder	☐	☐	☐	☐	☐	
Social Anxiety Disorder	☐	☐	☐	☐	☐	
Agoraphobia	☐	☐	☐	☐	☐	
Panic Disorder	☐	☐	☐	☐	☐	
Specific Phobia	☐	☐	☐	☐	☐	
Obsessive Compulsive Disorder	☐	☐	☐	☐	☐	
Posttraumatic Stress Disorder	☐	☐	☐	☐	☐	
Adjustment Disorder	☐	☐	☐	☐	☐	
Major Depressive Disorder	☐	☐	☐	☐	☐	
Substance Use Disorder	☐	☐	☐	☐	☐	
Bipolar Disorder	☐	☐	☐	☐	☐	
Psychotic Disorder (e.g., Schizophrenia)	☐	☐	☐	☐	☐	
Eating Disorder	☐	☐	☐	☐	☐	
Personality Disorder	☐	☐	☐	☐	☐	
Autism Spectrum Disorder	☐	☐	☐	☐	☐	
Other: ${q://QID11/ChoiceTextEntryValue/15}	☐	☐	☐	☐	☐	

Part 4: Demographics

Thank you for your responses.

The next set of questions will ask for demographic information.

This helps us understand the needs and experiences of different people when seeking help for their anxiety. As with the previous questions, this information also helps us know how well our results apply to the wider population.

Your answers will not be used to identify you specifically.

What is your age?

- ○ 18 - 24 years
- ○ 25 - 34 years
- ○ 35 - 44 years
- ○ 45 - 54 years
- ○ 55 - 64 years
- ○ 65+ years

How do you describe your ethnicity? (e.g., "I am Aboriginal", "Caucasian", "I am of Chinese background", "I am Maori and Caucasian")

____

How do you describe your gender identity? Select all that apply.

- ☐ Male
- ☐ Female
- ☐ Non-binary / third gender
- ☐ __________________ Prefer to self describe (please specify)
- ☐ Prefer not to say

Part 5: Overall Reflections

Thank you.

You will now be asked for some final reflections on the experience of seeing your GP for anxiety.

Overall, what was good about your experience of seeking help from your GP?

What was bad about your experience, or could have been done better?

Do you have any other suggestions about how we can improve care for anxiety in Australia?

End of Survey

Almost finished!

Thank you for participating in our survey.

Please click "submit" below to send your responses to the researcher.

Powered by Qualtrics
